# Physical Training in the Schools

**Published:** 1917-02

**Authors:** 


MRS. F. E. DANIEL, Publisher and Managing Editor.

On account of the editor's inability to secure red paper for the cover pages of the JOURNAL, it appears this month for the first time, in its history, perhaps, in other than a red cover. However, the arrangement is only a temporary one, and the red cover will be resumed just as soon as it can be obtained.

### Physical Training in the Schools.

The leading educators of the country have been aroused to the importance of more systematic physical training in the schools by a statement recently made by President Wilson to the effect that "physical training is needed, but can be had without compulsory military service." This has been construed as meaning that the President is opposed to compulsory military training or optional military training in the public schools followed by compulsory military service to compensate the government for its military education. There are many people who conscientiously oppose training the youth of a country to fight, but all agree that there should be some systematic physical training for children of both sexes.

That the schools have not given sufficient attention to the physical development of youth is generally agreed. It has for ages been admitted that physical disability is a severe handicap to mental development, but the schools have not seriously considered plans for securing the highest physical development. Therefore a committee of educators has entered upon a definite plan for preparing youth for all the demands of citizenship through physical training of a kind that will prepare both boys and girls for the pursuits of peace or the vicissitudes of war.

To accomplish this, bills will be presented to the Legislatures of every State for a systematic establishment of physical education and training in the public schools. Since the physicians, more than any other class, realize the great importance of proper physical development, it is hoped and believed that they will interest themselves in the measure wherever it is proposed.

No elaborate machinery is to be suggested, no difficult system of instruction, but realizing that the best time to begin physical instruction and training is in childhood, more attention will, under the measure proposed, be given to physical education in the primary and high schools. As a rule, the universities and colleges that are State supported are already giving a fair share of attention to physical culture, but heretofore it has been woefully neglected in the lower schools of most States. The proposition certainly affords fine opportunity for progressive physicians to help along a movement that should do incalculable good.

